# Comparison of 1-Year Health Care Costs and Use Associated With Open vs Robotic-Assisted Radical Prostatectomy

**DOI:** 10.1001/jamanetworkopen.2021.2265

**Published:** 2021-03-22

**Authors:** Kennedy E. Okhawere, I-Fan Shih, Shih-Hao Lee, Yanli Li, Jaime A. Wong, Ketan K. Badani

**Affiliations:** 1 Department of Urology, Icahn School of Medicine at Mount Sinai, New York, New York; 2 Intuitive Surgical Inc, Sunnyvale, California; 3 Veterans Affairs Palo Alto Healthcare System, Palo Alto, California

## Abstract

**Importance:**

With the current patterns of adoption and use of robotic surgery and improvement in the overall survival of patients with prostate cancer, it is important to evaluate the immediate and long-term cost implications of treatments for patients with prostate cancer.

**Objective:**

To compare health care costs and use 1 year after open radical prostatectomy (ORP) vs robotic-assisted radical prostatectomy (RARP).

**Design, Setting, and Participants:**

This retrospective cohort study used a US commercial claims database from January 1, 2013, to December 31, 2018. A total of 11 457 men aged 18 to 64 years who underwent inpatient radical prostatectomy for prostate cancer and were continuously enrolled with medical and prescription drug coverage from 180 days before to 365 days after inpatient prostatectomy were identified. An inverse probability of treatment weighting analysis was performed to examine the differences in costs and use of health care services by surgical modality. Data analysis was conducted from September 2019 to July 2020.

**Exposures:**

Type of surgical procedure: ORP vs RARP.

**Main Outcomes and Measures:**

Three outcomes within 1 year after the inpatient prostatectomy were investigated: (1) total health care costs, including reimbursement paid by insurers and out of pocket by patients; (2) health care use, including inpatient readmission, emergency department, hospital outpatient, and office visits; and (3) estimated days missed from work due to health care use.

**Results:**

Of the 11 457 patients who underwent inpatient prostatectomy, 1604 (14.0%) had ORP and 9853 (86.0%) had RARP and most patients (8467 [73.9%]) were aged 55 to 64 years. Compared with patients who underwent ORP, those who received RARP had a higher cost at the index hospitalization (mean difference, $2367; 95% CI, $1821-$2914; *P* < .001), but similar total cumulative costs were observed within 180 days (mean difference, $397; 95% CI, −$582 to $1375; *P* = .43) and 1 year after discharge (−$383; 95% CI, −$1802 to $1037; *P* = .60). One-year postdischarge health care use was significantly lower in the RARP compared with ORP group for mean numbers of emergency department visits (−0.09 visits; 95% CI, −0.11 to −0.07 visits; *P* < .001) and hospital outpatient visits (−1.5 visits; −1.63 to −1.36 visits; *P* < .001). The reduction in use of health care services among patients who underwent RARP translated into additional savings of $2929 (95% CI, $1600-$4257; *P* < .001) and approximately 1.69 fewer days (95% CI, 1.49-1.89 days; *P* < .001) missed from work for health care visits.

**Conclusions and Relevance:**

Total cumulative cost in this study was similar between ORP and RARP 1 year post discharge; this finding suggests that lower postdischarge health care use after RARP may offset the higher costs during the index hospitalization.

## Key Points

**Question:**

Is robotic-assisted radical prostatectomy associated with lower 1-year health care cost and use compared with open radical prostatectomy surgery?

**Findings:**

In this economic evaluation study using a US claim database of 11 457 patients undergoing radical prostatectomy, health care use was significantly lower after robotic-assisted compared with open radical prostatectomy. Total cumulative cost was similar between the groups 1-year post discharge.

**Meaning:**

These findings suggest that lower postdischarge health care use after robotic-assisted prostatectomy may offset the higher costs during the index hospitalization.

## Teaser

This cohort study examines the use of health care services and cost at the index hospitalization and 1 year post discharge in men undergoing open vs robotic-assisted radical prostatectomy.

## Introduction

Prostate cancer is the most commonly diagnosed cancer in men older than 50 years in the US,^1,2^ accounting for about one-fifth of all new cancers in men in 2019.^3^ Since the development of prostate-specific antigen testing and improvement in treatment strategies, the incidence and early detection of organ-confined prostate cancer have increased, with an increase in the 5-year survival rate.^4,5^ The overall decrease in mortality, with an increase in the prevalence of prostate cancer,^6^ underscores the importance of evaluating the immediate and long-term cost implications of treatments for patients with prostate cancer and their effects in the health care system.

Although the treatment approach for prostate cancer varies by geographic location, cancer stage, and available technology,^7^ over the years there has been a shift of surgical management of prostate cancers to more minimally invasive methods, such as laparoscopic surgeries and, more recently, robotic-assisted surgeries.^8^ Although open surgery is still common for managing prostate cancer, robotic-assisted prostatectomy is rapidly becoming the most frequently used approach in the US.^9,10^ As of 2012, the use of a robotic approach was 73%, indicating a shift in surgical management and use of a robotic-assisted approach.^11^

Studies comparing the perioperative benefit of robotic-assisted prostatectomy vs open surgery have reported reduced blood loss, shorter length of hospital stay, fewer intraoperative and postoperative complications, and better functional outcomes.^1,12,13,14^ However, opting for robotic surgery based on these benefits comes at a cost. The robotic-assisted approach is often associated with a higher cost of acquisition, and maintenance, and the purchase of disposables used as an adjunct during surgery,^15^ which is reflected in the immediate perioperative cost of the surgical procedure. Comparing the costs of laparoscopic and robotic-assisted prostatectomies with open prostatectomies, Lotan et al^15^ reported that open prostatectomies were the least expensive and most cost-effective approach to prostatectomy. They further elucidated that there was a cost advantage for open prostatectomy over the robotic-assisted approach, even when the initial cost of purchasing the robot was not factored in. Conversely, another study reported that the robotic approach may not be more costly in all care settings, and cost may differ by hospital type and setting and might be dependent on the length of stay and local hospitalization cost.^16^

The long-term benefits of the robotic-assisted approach may offset the cost implication in the use of robotic surgery compared with the open approach if the cost of postoperative health care is properly evaluated (eg, prolonged hospital stay and cost of readmissions, management of postoperative complications, and the care setting). Numerous studies have reported on the immediate perioperative cost of the robotic approach,^10^ but few have reported on the long-term cost comparison between the 2 approaches.^9^ Hence, the goal of this study was to compare the long-term cost and health care use after prostatectomy between a robotic-assisted radical prostatectomy (RARP) and an open radical prostatectomy (ORP).

## Methods

### Data Source

We conducted a retrospective data analysis using an aggregated database (IBM MarketScan Commercial Claims and Encounter Database) that includes paid health care claims of millions of employees and their dependents from approximately 350 private sector payers in the US. The database captures person-specific information on annual insurance enrollment along with inpatient, outpatient, and prescription drug services from selected large employers and government and public organizations with a range of employer-provided health insurance plans. Because this was an observational study of deidentified patients in the database without the possibility for reidentification, institutional review board approval was not required in accordance with the Health Insurance Portability and Accountability Act privacy rule. This study followed the Consolidated Health Economic Evaluation Reporting Standards (CHEERS) reporting guideline where appropriate.

### Study Population

All men between ages 18 and 64 years who had undergone inpatient radical prostatectomy for prostate cancer between January 1, 2013, and December 31, 2018, were identified. We used *International Classification of Diseases and Related Health Problems, Ninth Revision* (*ICD-9*) and *International Statistical Classification of Diseases, Tenth Revision* (*ICD-10*) and *Current Procedural Terminology* to define the eligible prostatectomy cases and differentiate surgical approaches (eTable 1 in the Supplement). To be eligible for data analysis, patients were required to be continuously enrolled with medical and prescription drug coverage from 180 days before to 365 days after inpatient prostatectomy. Exclusion criteria included (1) inpatient cases that were not coded with diagnosis-related group codes 707 or 708, (2) metastatic cancer cases, (3) pure laparoscopic approach owing to a very small number of cases, (4) discharges with extreme total payment in index hospitalization (<1% or >99%), and (5) negative payments for baseline or follow-up total payment. Data analysis was conducted from September 2019 to July 2020.

### Outcomes

Our study investigated 3 main outcome measures within 1 year after the inpatient prostatectomy: (1) total health care cost, including reimbursement paid by insurers and out of pocket by patients; (2) health care use, including inpatient readmission, emergency department, hospital outpatient, and office visits; and (3) estimated days missed from work due to health care use. Total cost was calculated by adding facility and professional payments during the inpatient stay (index surgery) and all health services–related payments within 1 year after discharge, including inpatient, outpatient, and prescription drug claims. Based on place of service codes and descriptions, we differentiated the outpatient services into emergency department, hospital outpatient, and office visits. Patients who did not have insurance claims during the follow-up period were assumed to incur 0 health care cost and use. Total costs were inflation adjusted to 2018 US dollars using the general consumer price index. If a patient had a claim for an inpatient service, we directly converted the length of stay to days of health care use. To estimate the number of days missed from work to have health care visits, we assumed a half-day of use for an office visit claim and a full day of use for claims related to emergency department, urgent care facility, or other hospital outpatient visit.^17,18^ All of the above factors were summed to derive the estimated days missed from work due to health care use for each patient.

### Patient Factors

The covariates were selected based on prior knowledge and literature.^17,18,19^ Patient-level baseline sociodemographic and clinical characteristics included age, sex, region, insurance plan, metropolitan vs nonmetropolitan area, annual income level (based on median income within an area code), and year of surgery. Insurance plans were classified into capitated plan, comprehensive insurance, preferred provider organization, noncapitated point of service, and other insurance plans. We calculated the Charlson Comorbidity Index score (excluding prostate cancer) and identified lymph node dissection and current or previous smokers using the *ICD-9* and *ICD-10* diagnosis codes presented at the index hospitalization and in the 180-day preoperative period.

### Statistical Analysis

Patient characteristics at baseline were compared between the ORP and RARP groups using χ^2^ tests for categorical and binary variables and Kruskal-Wallis test for nonnormally distributed variables. To minimize the effect of potential confounding factors without reducing the size of the study population, we performed inverse probability of treatment weighting (IPTW) using stabilized weights to generate a single pseudopopulation and estimated the unbiased average treatment effects.^20^ Inverse probability of treatment weights were calculated as the inverse of patients’ estimated probability of surgical approach using a logistic regression model with all the baseline patient factors. We applied a generalized linear model weighted by the IPTW values and adjusted for the total health care costs in the 180-day preoperative period (ie, baseline cost) to estimate the index hospitalization and postdischarge health care costs using γ distribution and services use with logistic regression. We also compared the mean inpatient length of stay during readmission and the mean number of emergency department, hospital outpatient, and physician office visits. We performed a goodness-of-fit test and modified Park test, and the best model was defined by the minimum Akaike information criterion. Costs, hospital outpatient visits, office visits, and estimated days missed from work were estimated using γ distribution; emergency department and inpatient services were modeled using 0-inflated Poisson regression. We further used the service subcategory code variable in the database to identify the detailed service type in outpatient claims. In a sensitivity analysis, we extended the follow-up period to 2 years to explore the health care costs in a longer time frame. The additional cost analyses were conducted for patients who had 2-year continuous insurance enrollment after the index surgery. To evaluate the association between outliers and costs, we further included the cases discharged with extreme total payment in the index hospitalization (<1% or >99%). All analyses were performed using SAS software, version 9.4 (SAS Institute Inc). A 2-tailed value of *P* < .05 was considered statistically significant.

## Results

**Figure 1.**
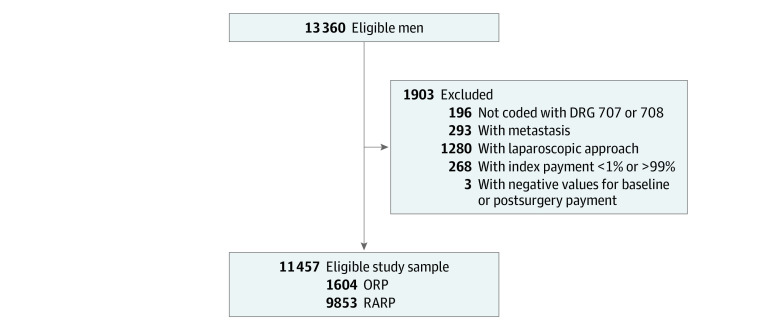
Flow Diagram of Study DRG indicates diagnosis-related group; ORP, open radical prostatectomy; and RARP, robotic-assisted radical prostatectomy.

A total of 13 360 men who underwent inpatient radical prostatectomy for prostate cancer and had 1-year continuous insurance enrollment were identified between 2013 and 2018. After applying exclusion criteria, the final cohort consisted of 11 457 patients: 1604 men (14.0%) underwent ORP and 9853 (86.0%) underwent RARP (Figure 1). Most patients (9662 [84.3%]) had a Charlson Comorbidity Index score of 1 or higher; of these, 8467 (73.9%) men were aged between 55 and 64 years. The preferred provider organization plan was the most common insurance plan (6663 [58.2%]) and 4920 (42.9%) patients lived in the southern region of the US. Descriptive data stratified by surgical approach are summarized in Table 1. Patients undergoing ORP were younger and more likely to have lymph node dissections during inpatient prostatectomy or surgery compared with those who underwent RARP. Significantly greater proportions of patients who received RARP lived in the North Central region of the US, metropolitan areas, and regions with higher average annual incomes. All the baseline sociodemographic variables were balanced after applying IPTW.

**Table 1.** Baseline Demographic Characteristics of Patients Undergoing ORP or RARP Before and After Inverse Probability of Treatment Weighting Adjustment

Variable	IPTW, No. (%)						
	Before				After		
	All (n = 11457)	ORP (n = 1604)	RARP (n = 9853)	*P* value	ORP (n = 1604)	RARP (n = 9853)	*P* value
Age, y							
18-44	160 (1.4)	27 (1.7)	133 (1.3)	.04	24 (1.5)	137 (1.4)	.95
45-54	2830 (24.7)	358 (22.3)	2472 (25.1)		393 (24.5)	2433 (24.7)	
55-64	8467 (73.9)	1219 (76.0)	7248 (73.6)		1184 (73.8)	7282 (73.9)	
Income/y							
>$40 000	2253 (19.7)	243 (15.1)	2010 (20.4)	<.001	301 (18.8)	1936 (19.6)	.49
Insurance plan							
Capitated	1337 (11.7)	190 (11.8)	1147 (11.6)	.007	188 (11.7)	1150 (11.7)	>.99
Comprehensive	581 (5.1)	77 (4.8)	504 (5.1)		78 (4.9)	499 (5.1)	
Noncapitated POS	810 (7.1)	147 (9.2)	663 (6.7)		119 (7.4)	697 (7.1)	
PPO	6663 (58.2)	932 (58.1)	5731 (58.2)		927 (57.8)	5728 (58.1)	
Others	1921 (16.8)	240 (15.0)	1681 (17.1)		268 (16.7)	1652 (16.8)	
Unknown	145 (1.3)	18 (1.1)	127 (1.3)		20 (1.2)	124 (1.3)	
Nonmetropolitan status	1631 (14.2)	301 (18.8)	1330 (13.5)	<.001	224 (14.0)	1404 (14.2)	.80
Region							
North Central	2903 (25.3)	327 (20.4)	2576 (26.1)	<.001	401 (25.1)	2496 (25.3)	>.99
Northeast	2031 (17.7)	267 (16.6)	1764 (17.9)		280 (17.5)	1746 (17.7)	
South	4920 (42.9)	807 (50.3)	4113 (41.7)		693 (43.2)	4231 (42.9)	
West	1508 (13.2)	178 (11.1)	1330 (13.5)		212 (13.2)	1297 (13.2)	
Unknown	95 (0.8)	25 (1.6)	70 (0.7)		12 (0.7)	81 (0.8)	
Procedure year							
2013	2291 (20.0)	399 (24.9)	1892 (19.2)	<.001	321 (20.0)	1969 (20.0)	>.99
2014	2679 (23.4)	411 (25.6)	2268 (23.0)		375 (23.4)	2304 (23.4)	
2015	2628 (22.9)	346 (21.6)	2282 (23.2)		363 (22.6)	2259 (22.9)	
2016	2027 (17.7)	250 (15.6)	1777 (18.0)		287 (17.9)	1744 (17.7)	
2017	1832 (16.0)	198 (12.3)	1634 (16.6)		254 (15.8)	1575 (16.0)	
Tobacco abuse/history	922 (8.0)	125 (7.8)	797 (8.1)	.72	131 (8.2)	793 (8.0)	.85
Charlson comorbidity							
0	1795 (15.7)	982 (61.2)	6059 (61.5)	.45	988 (61.6)	6055 (61.4)	.86
1	7041 (61.5)	355 (22.1)	2266 (23.0)		371 (23.1)	2255 (22.9)	
≥2	2621 (22.9)	267 (16.6)	1528 (15.5)		242 (15.1)	1542 (15.7)	
Lymph node dissection	5270 (46.0)	794 (49.5)	4476 (45.4)	.003	732 (45.6)	4529 (46.0)	.88
Baseline payment							
Median (IQR)	6939 (4466-11 197)	6804 (4261-11 124)	6954 (4498-11 207)	.23	NA	NA	NA
Mean (SD)	9806 (12 615)	9896 (14 769)	9792 (12 229)	.76	NA	NA	NA

Abbreviations: IPTW, inverse probability of treatment weighting; IQR, interquartile range; NA, not applicable; ORP, open radical prostatectomy; RARP, robotic-assisted radical prostatectomy; POS, point of service; PPO, preferred provider organization.

**Figure 2.**
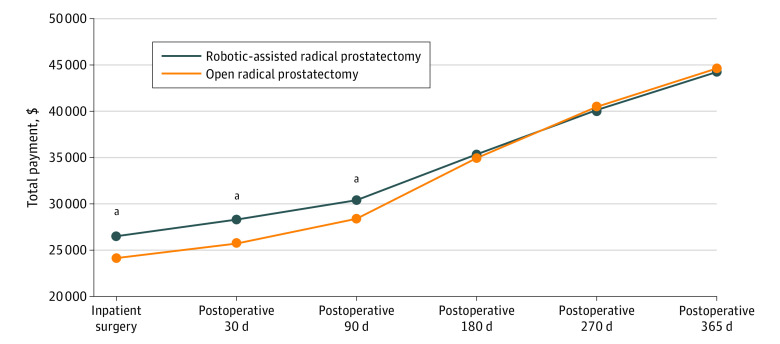
Time Series Graphics for the Inverse Probability of Treatment Weighting–Adjusted Payment Total cost was calculated by adding facility and professional payments during the inpatient stay (index surgery) and all health services–related costs within 1 year after discharge, including inpatient, outpatient, and prescription drug services cumulatively. ^a^*P* < .001.

The mean (SD) health care cost within 180 days before the index operation was similar between the RARP vs ORP groups ($9792 [$12 229] vs $9896 [$14 769]; *P* = .76). eTable 2 in the Supplement and Figure 2 illustrate the unadjusted and IPTW-adjusted total health care cost patterns over time. For the index inpatient prostatectomy, IPTW-adjusted cost was significantly higher for RARP vs ORP ($26 504; 95% CI, $26 284-$26 726 vs $24 137; 95% CI, $23 642-$24 642; *P* < .001), resulting in a cost difference of $2367 (95% CI, $1821-$2914; *P* < .001) per prostatectomy. The adjusted cost differences between the 2 groups gradually decreased over time and became nonsignificant 180 days post discharge (mean difference, $397; 95% CI, −$582 to $1375; *P* = .43). The adjusted 1-year total health cost remained comparable between RARP and ORP at the 1-year follow-up point (mean difference, −$383; 95% CI, −$1802 to $1037; *P* = .60). Data on the use of health care services within 365 days after the index inpatient surgery are listed in Table 2.

**Table 2.** Adjusted Differences in 1-Year Postindex Cost, Health Care Use, and Estimated Days Missed From Work Between Surgical Modalities

Variable	ORP		RARP		Adjusted differences (ORP as reference)		*P* value
	Mean (95% CI)	No. (%)	Mean (95% CI)	No. (%)	Mean (95% CI)	No. (%)	
Total payment, $^a^	20 276 (19 058-21 571)	NA	17 347 (16 919-17785)	NA	−2929 (−4257 to −1600)	NA	<.001
Readmission	NA	147.75 (9.2)	NA	829.09 (8.4)	NA	0.90 (0.75-1.08)	.25
Inpatient LOS, d	0.39 (0.32-0.46)	NA	0.40 (0.38 to 0.43)	NA	0.01 (−0.02 to 0.05)	NA	.70
ED visit	NA	447.67 (28.0)	NA	2483.92 (25.2)	NA	0.87 (0.77-0.98)	.02
No. of visits							
ED	0.47 (0.42-0.51)	NA	0.38 (0.36-0.39)	NA	−0.09 (−0.11 to −0.07)	NA	<.001
Hospital outpatient	NA	1253.91 (78.3)	NA	7524.21 (76.4)	NA	0.89 (0.78-1.01)	.06
Outpatient	7.18 (7.05-7.31)	NA	5.68 (5.63-5.72)	NA	−1.5 (−1.63 to −1.36)	NA	<.001
Office	13.54 (13.36-13.72)	NA	13.32 (13.25-13.39)	NA	−0.22 (−0.41 to −0.03)	NA	.02
No. of days off	14.82 (14.63-15.00)	NA	13.12 (13.05-13.20)	NA	−1.69 (−1.89 to −1.49)	NA	<.001

Abbreviations: ED, emergency department; LOS, length of stay; NA, not applicable; ORP, open radical prostatectomy; RARP, robotic-assisted radical prostatectomy.

^a^ Total payment included health services payments after discharge without index surgery payment.

Although we observed similar readmission rates with ORP (9.2%) and RARP (8.4%) (*P* = .25), patients who underwent RARP were less likely than those who underwent ORP to visit the emergency department (25.2% vs 28.0%; *P* = .02) and hospital outpatient department (76.4% vs 78.3%; *P* = .06) in the postsurgery period. Patients in the RARP group also had fewer mean emergency department visits (−0.09; 95% CI, −0.11 to −0.07; *P* < .001), fewer mean hospital outpatient visits (−1.5; 95% CI, −1.63 to −1.36; *P* < .001), and fewer office visits (−0.22; 95% CI, −0.41 to −0.03; *P* = .02). The reduction in health care use among patients in the RARP group translated into additional savings of $2929 (95% CI, $1600-$4257; *P* < .001) and approximately 1.69 days (95% CI, 1.49-1.89; *P* < .001) fewer days missed from work due to health care visits over the 1-year postdischarge period. The breakdown of outpatient costs showed therapeutic radiologic and facility procedure expenditures were the 2 highest service categories (eTable 3 in the Supplement). Use of RARP was associated with approximately $2000 less outpatient therapeutic radiologic expenditures during the postsurgery period compared with ORP.

An extended analysis was conducted to assess the total health care costs during a longer time after the index surgery (eFigure in the Supplement). Restricting the analysis to patients who had 2 years or more continuous enrollment after discharge (n = 6929) did not substantially alter the 1-year follow-up finding. Total health care costs within 2 years were higher for ORP vs RARP ($59 856 vs $57 687), resulting in a nonsignificant cost difference of $2169 (*P* = .10). The results of additional analysis including cases with outlier costs remained similar (eTable 4 in the Supplement).

## Discussion

Technologies such as robotic-assisted surgery are increasingly being accepted and implemented in health care settings. By 2017, there was a reported 32% year-over-year growth in the use of robotic surgery systems among general surgeons.^21^ As prostate cancer continues to be one of the most commonly diagnosed cancers in men older than 50 years in the US, the evaluation of immediate and long-term cost implications between RARP and ORP for prospective patients should be examined in further detail. More specifically, cost analysis of RARP vs ORP should account for long-term savings instead of only initial up-front costs. In this study, we compared the health care cost and use of 11 457 patients who underwent radical prostatectomy using a large cohort of national private insurance data. We found that RARP had a higher cost at the index hospitalization, but similar total cumulative costs were observed between the RARP and ORP groups at 180 and 365 days after surgery. One-year postdischarge health care use was significantly lower in the RARP compared with ORP group for emergency department and hospital outpatient visits. The reduction in health care use among patients who underwent RARP translated into an additional savings of $2929 and approximately 1.69 fewer days missed from work. We also found that RARP resulted in an approximately $2000 lower cost in postoperative outpatient therapeutic radiologic expenditures.

Similar to our findings, studies comparing costs between RARP and ORP have reported a higher cost of index surgery for RARP compared with ORP.^10^ For example, Kim et al^19^ evaluated the total cost attributable to RARP and ORP and reported that, despite the benefit of a shorter hospital stay associated with the use of RARP, there was a significantly higher cost attributable to RARP after controlling for patient- and hospital-related factors. Although this finding is true for most studies, a longitudinal assessment of the cost implication of RARP is important, especially considering the likelihood of higher postoperative morbidity associated with ORP. Extending beyond the index surgery, Leow et al^22^ reported a higher cost related to RARP 90 days after surgery, attributing most of these costs to the operating room and surgical supplies. This finding is consistent with the significantly higher costs of the index surgery and cumulative cost at 90 days in our study. In a retrospective analysis conducted in England, Hughes et al^23^ reported a significantly higher postoperative cost incurred by patients who had ORP compared with RARP at 360 days and 1080 days post surgery. Compared with this study, our study reported a nonsignificant difference between RARP and ORP from 180 days after surgery. Perhaps this variation is because our study accounted for the baseline differences in patients’ health care use by IPTW. In addition, the discrepancy may be due to differences in country-related health care costs. Other studies by Niklas et al^24^ and Bijlani et al^14^ reported lower postoperative health care costs for RARP and similar equivalent costs between ORP and RARP at 2 years^24^and 3 years.^14^ In the eFigure in the Supplement, we extrapolated our findings beyond 1 year and found equivalence between the cost of the 2 surgical approaches up to 2 years, with a nonstatistically significant change in favor of lower costs for RARP. Although the difference was not significant, the change suggests potential cost savings ($2169; *P* = .10) for RARP that, if further extrapolated, might reach a significant level when patients are followed up for a longer time, such as 1080 days. We also posit that, from a societal perspective, there will be additional health care cost savings for patients who undergo RARP because they have fewer missed days from work and reduced health care use owing to the associated morbidity related to the procedure that is less than the morbidity with ORP. The reduced number of days missed from work has been reported in a previous study.^25^ We limited our sensitivity analysis to 2 years because we did not have a sufficient sample size and follow-up time to analyze a longer period. Beyond 2 years, we assumed that postoperative costs may not be independent of other competitive risk factors that may increase the cost of health care use between the groups, hence limiting the estimation of cost for a longer follow-up period.

Concerning health care use, several studies have reported less health care use postoperatively among patients who have undergone RARP compared with ORP.^8,9,23,24^ Niklas et al^24^ reported that patients who underwent RARP had significantly lower hospital readmission rates and complications compared with patients who underwent ORP. Although the lower rate of readmission for RARP in our study was not significantly different from that for ORP, our finding is similar to the one reported by Niklas et al. Hughes et al^23^ also reported that men who had RARP had fewer inpatient readmissions, hospital bed-days, and excess bed-days at 360 and 1080 days. Hyldgård et al,^9^ in a study evaluating the cost attributable to RARP, also reported that patients who had undergone ORP had marginally higher primary care use in the period up to 12 months after surgery. They further concluded that the net effect of RARP on the health care resources used was not significant. While assessing the breakdown of postoperative outpatient services costs, we found a higher expenditure on therapeutic radiologic procedures for patients who had ORP. This higher cost was noted in a study by Gandaglia et al,^8^ with more patients who underwent ORP vs RARP requiring radiotherapy sessions postoperatively (10.0% vs 7.2%), which might be associated with higher costs compared with RARP. A higher rate of postoperative positive surgical margin has been shown to be associated with ORP in a previous study^24^; thus, patients who undergo ORP may be at increased risk of local relapse and would benefit from adjuvant radiotherapy, which may contribute to the cost of health care use after the index surgery.^26^ Although our study did not account for the difference with regard to perioperative outcomes owing to the lack of clinical data, in this context the use of additional treatments postoperatively between the 2 surgical approaches in a similar population may be attributed to the equivalence and balance of cost over a protracted period.

### Strengths and Limitations

Most studies evaluating and comparing cost after prostatectomies are limited by their methodologies, model assumptions, and lack of adjustment of patients’ baseline difference.^10^ Apart from the fact that our study is based on a large US commercial database that captures longitudinal payments after the index surgery, one of the strengths of this study is that we were able to describe health care service use after surgery and evaluate the change of health care cost over 1 year, adjusting for patients' baseline differences up to 180 days before index surgery.

Our study also has limitations. The retrospective design makes it difficult to account for possible unknown confounders. Given that this was an actual claims database, there is a potential risk for errors in data coding with patient identification and data extraction. Also, the results of this study may not be generalizable to older, Medicare, Medicaid, or uninsured patients. We recognize the absence of measurement and adjustment for race/ethnicity, stage of cancer, hospital characteristics (eg, lack of robotic machines and patient volume), surgeon characteristics (eg, skill level), and metastatic disease as other factors that might influence the decision for surgical modality. Apart from using IPTW to account for some of the variability that may exist, we adjusted for lymph node dissection as a factor in our IPTW. However, this method may not fully adjust for the differences or unknown confounders that may exist between ORP and RARP. In addition, days missed from work for health care visits is a proxy for the time off work for seeking follow-up visits and not truly lost work productivity.

## Conclusions

The results of this study suggest that the total cumulative health care cost is equivalent when comparing ORP and RARP procedures 1 year after surgery. Robotic may be associated with lower postdischarge health care use, which may offset the higher costs during the index hospitalization.

## Notes

Supplement. **eTable 1.** Diagnosis and Procedure Codes Used to Determine Eligibility **eTable 2.** Breakdown of Outpatient Services Costs **eTable 3.** Unadjusted Costs Between Surgical Modalities **eTable 4.** Adjusted Costs Between Surgical Modalities: Sensitivity Analysis Including Cases With Outlier Costs **eFigure.** Time Series Graphics for the IPTW-Adjusted Payment
