# Supplementary material for: Comparison of 1-Year Health Care Costs and Use Associated With Open vs Robotic-Assisted Radical Prostatectomy
Source: JAMA Netw Open. 2021 Mar 22;4(3):e212265. doi: 10.1001/jamanetworkopen.2021.2265 (PMC7985723; doi:10.1001/jamanetworkopen.2021.2265)
Supplement: Supplement. — eTable 1. Diagnosis and Procedure Codes Used to Determine Eligibility eTable 2. Breakdown of Outpatient Services Costs eTable 3. Unadjusted Costs Between Surgical Modalities eTable 4. Adjusted Costs Between Surgical Modalities: Sensitivity Analysis Including Cases With Outlier Costs eFigure. Time Series Graphics for the IPTW-Adjusted Payment [file jamanetwopen-e212265-s001.pdf]

Supplementary Online Content

Okhawere KE, Shih I-F, Lee S-H, Li Y, Wong JA, Badani KK. Comparison of 1-year health care costs and use associated with open vs robotic-assisted radical prostatectomy. *JAMA Netw Open*. 2021;4(3):e212265. doi:10.1001/jamanetworkopen.2021.2265

eTable 1. Diagnosis and Procedure Codes Used to Determine Eligibility

eTable 2. Breakdown of Outpatient Services Costs

eTable 3. Unadjusted costs Between Surgical Modalities

eTable 4. Adjusted Costs Between Surgical Modalities: Sensitivity Analysis Including Cases With Outlier Costs

eFigure. Time Series Graphics for the IPTW-Adjusted Payment

This supplementary material has been provided by the authors to give readers additional information about their work.

© 2021 Okhawere KE et al. *JAMA Network Open.*

eTable 1. Diagnosis and Procedure Codes Used to Determine Eligibility

**Diagnosis**	-
ICD-9	
185	Malignant neoplasm of prostate
ICD-10	
C61	Malignant neoplasm of prostate
**Procedure**	
ICD-9	
60.5	Radical prostatectomy
ICD-10	
0VT00ZZ	Resection of prostate, open approach
0VT04ZZ	Resection of prostate, percutaneous endoscopic approach
**Robotic**	
ICD-9	
17.41	Open robotic assisted procedure
17.42	Laparoscopic robotic assisted procedure
17.43	Percutaneous robotic assisted procedure
17.44	Endoscopic robotic assisted procedure
17.45	Thoracoscopic robotic assisted procedure
17.49	Other and unspecified robotic assisted procedure
ICD-10	
8E0W0CZ	Robotic assisted procedure of trunk region, open approach
8E0W3CZ	Robotic assisted procedure of trunk region, percutaneous approach
8E0W4CZ	Robotic assisted procedure of trunk region, percutaneous endoscopic approach
8E0W7CZ	Robotic assisted procedure of trunk region, via natural or artificial opening
8E0W8CZ	Robotic assisted procedure of trunk region, via natural or artificial opening endoscopic
8E0WXCZ	Robotic assisted procedure of trunk region
CPT/HCPCS	
55866	Laparoscopy, surgical prostatectomy, retropubic radical, including nerve sparing, includes robotic assistance, when performed
S2900	Surgical techniques requiring use of robotic surgical system (list separately in addition to code for primary procedure)

© 2021 Okhawere KE et al. *JAMA Network Open.*

eTable 2. Breakdown of outpatient services costs

	All (n=11457) Mean	ORP (n=1604) Mean	RARP (n=9853) Mean	ORP as reference Mean Diff
Radiology OP Therapeutic Radiology	$5,861	$7,887	$5,531	$-2,356
Facility OP Procedures	$1,711	$1,851	$1,689	$-162
Facility OP ER	$553	$685	$532	$-153
Physician Specialty OP Office Visits	$496	$518	$493	$-25
Physician Specialty OP Procedures	$491	$535	$484	$-52
Radiology OP CT Scans	$348	$530	$318	$-212
Facility OP Specialty Drugs	$342	$425	$329	$-96
Professional OP Specialty Drugs	$336	$287	$344	$57
Laboratory OP Chemistry Tests	$302	$354	$293	$-60
Physician Non-Specialty OP Office Visits	$301	$323	$298	$-25

ORP, open radical prostatectomy; RARP, robotic-assisted radical prostatectomy; Diff, difference; OP, outpatient; ER, emergency room.

© 2021 Okhawere KE et al. *JAMA Network Open.*

eTable 3. Unadjusted costs between surgical modalities

	ORP		RARP		Diff		P-value
	Mean	95% CI	Mean	95% CI	Mean	95% CI	
Index surgery	23924	23431-24426	26554	26332-26778	2630	2085-3175	<.0001
Index to 30-day post-discharge	25657	25109-26216	28371	28125-28619	2715	2108-3320	<.0001
Index to 90-day post-discharge	28696	28043-29364	30594	30312-30880	1898	1180-2617	<.0001
Index to 180-day post-discharge	35445	34505-36411	35730	35344-36120	285	-744-1314	0.59
Index to 270-day post-discharge	41225	40024-42463	40754	40271-41244	-471	-1784-842	0.48
Index to 365-day post-discharge	45925	44524-47370	45095	44535-45662	-830	-2361-700	0.29

ORP, open radical prostatectomy; RARP, robotic-assisted radical prostatectomy; Diff, difference

© 2021 Okhawere KE et al. *JAMA Network Open.*

eTable 4. Adjusted costs between surgical modalities: sensitivity analysis including cases with outlier costs

	ORP		RARP		Diff		P-value
	Mean	95% CI	Mean	95% CI	Mean	95% CI	
Index surgery	24497	23938-25068	26881	26633-27132	2385	1767-3002	<.0001
Index to 30-day post-discharge	26048	25441-26670	28649	28379-28923	2601	1928-3273	<.0001
Index to 90-day post-discharge	28704	28015-29410	30745	30446-31047	2041	1281-2800	<.0001
Index to 180-day post-discharge	35212	34262-36188	35710	35319-36105	498	-542-1538	0.35
Index to 270-day post-discharge	40710	39529-41928	40473	39997-40956	-237	-1529-1054	0.72
Index to 365-day post-discharge	44868	43525-46253	44617	44075-45166	-252	-1720-1217	0.74

ORP, open radical prostatectomy; RARP, robotic-assisted radical prostatectomy; Diff, difference

© 2021 Okhawere KE et al. *JAMA Network Open.*

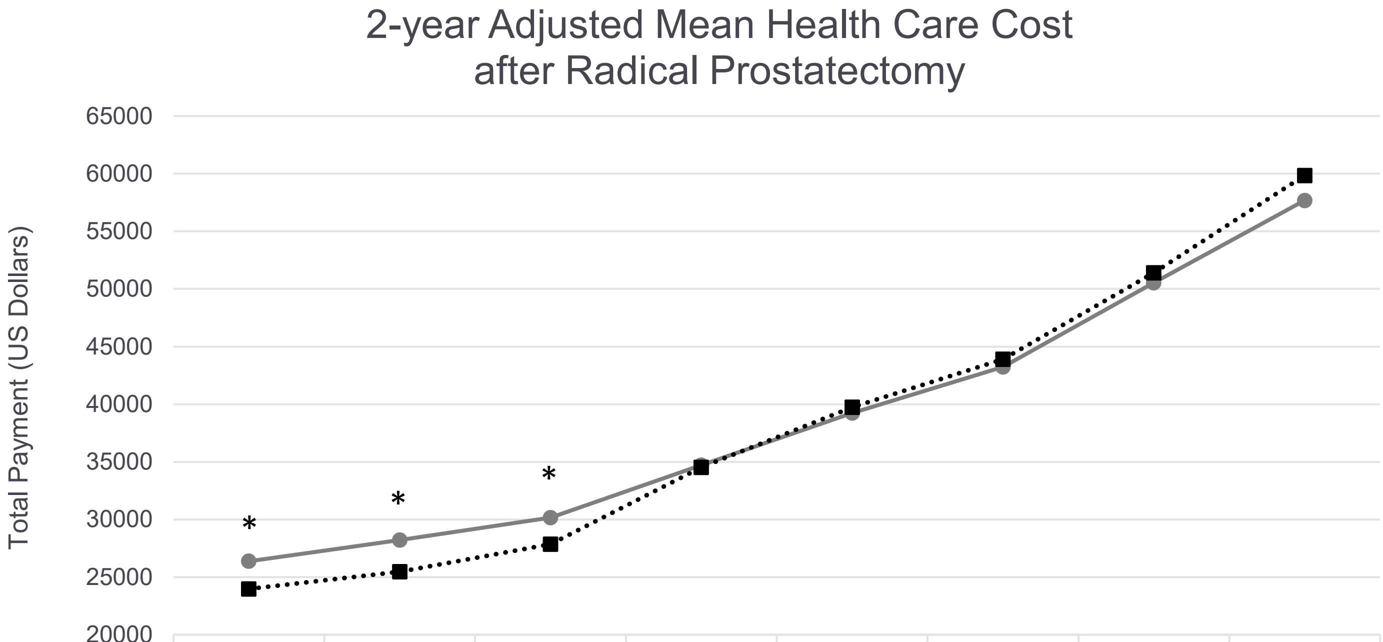

	inpatient surgery	post-op 30-day	post-op 90-day	post-op 180-day	post-op 270-day	post-op 365-day	post-op 545-day	post-op 730-day
Robotic	26375	28213	30170	34706	39228	43233	50543	57687
Open	23978	25481	27860	34517	39743	43896	51413	59856

eFigure. Time series graphics for the IPTW-adjusted payment. Total payment was calculated by adding facility and professional payments during the inpatient stay (index surgery) and all health services related payments within the 2-year after discharge, including inpatient, outpatient, and prescription drug claims cumulatively among patients who had 2-year insurance enrollment. * p<0.05

© 2021 Okhawere KE et al. *JAMA Network Open.*
